# The potential role of Colchicine in preventing coronary vascular disease in childhood‐onset lupus: a new view on an old drug

**DOI:** 10.1186/s12969-021-00504-6

**Published:** 2021-02-16

**Authors:** Dori Abel, Stacy P. Ardoin, Mark Gorelik

**Affiliations:** 1 Department of Pediatrics, NewYork-Presbyterian Hospital, Columbia University Irving Medical Center, 630 W. 168th Street, New York, NY 10032-3702 USA; 2 Department of Medicine, Division of Rheumatology and Immunology, The Ohio State University, 370 W. 9th Ave, Columbus, OH 43210 USA; 3 Department of Rheumatology, Nationwide Children’s Hospital, 700 Children’s Dr, Columbus, OH 43205 USA; 4 Department of Pediatrics, Division of Allergy, Immunology, and Rheumatology, Columbia University Irving Medical Center, 630 W. 168th St, New York, NY 10032-3702 USA

**Keywords:** Systemic lupus erythematous, Cardiovascular Disease, Colchicine, Atherosclerosis, Prevention

## Abstract

**Background:**

Patients with systemic lupus erythematous have a significantly increased risk of cardiovascular disease, which is not fully explained by traditional cardiovascular disease risk factors. Despite increasing life expectancy in patients with systemic lupus erythematous, mortality due to cardiovascular disease, the major cause of death in these patients, has not changed. Children with lupus suffer from more aggressive disease compared to their adult counterparts, and there is a growing concern for their increased risk of cardiovascular disease as they age.

**Body:**

There is an unmet need for therapies to address the increased risk of cardiovascular disease in childhood-onset lupus. Colchicine has many anti-inflammatory and cardiovascular protective properties, including inhibition of IL-1β and IL-18 activity, key proinflammatory cytokines that are predictive of future adverse cardiovascular events. In the Colchicine Cardiovascular Outcomes Trial (COLCOT), colchicine was recently found to have significant benefit with minimal risk in adults with previous myocardial infarction for prevention of secondary vascular disease. While adult studies are promising, no studies have been conducted in pediatric patients to investigate colchicine’s potential for cardiovascular protection in children and adolescents with lupus.

**Conclusions:**

Studies investigating colchicine’s potential role for cardiovascular protection are needed in pediatric patients with systemic lupus erythematous.

## Background

Due to advances in diagnosis and management of systemic lupus erythematous (SLE), patients are living longer. There is now increased focus on detection and prevention of long-term complications and downstream associated comorbidities in SLE, including atherosclerosis [1]. While all-cause mortality in patients with SLE has declined since 1975, mortality due to cardiovascular disease (CVD), the major cause of death in these patients, has remained stagnant [2]. One hypothesis for this discordance is that increased life expectancy in patients with SLE has led to more patients ultimately suffering from CVD [3]. Although atherosclerosis worsens with age, increased life expectancy does not fully explain this unchanged cardiovascular mortality [4]. Women under age 45 with SLE are over 50 times more likely to suffer a myocardial infarction (MI) than women of similar age in the Framingham Offspring Study [4], suggesting other factors than age contribute to CVD mortality in lupus patients. CVD is also significantly more common in young premenopausal women with SLE compared to controls [4].

Compared to their adult counterparts, children with SLE have more aggressive disease, a lower life expectancy, longer disease burden, higher incidence of lupus nephritis and neurological manifestations, and there is growing concern for their increased risk of CVD as they age [5–8]. Indeed, adults with history of childhood-onset SLE have a standardized mortality ratio of 2.5, with CVD the most common reported cause of death, and they experience these complications at an early age, with average age of first MI as early as 32 [7, 9]. Both traditional Framingham CVD risk factors and SLE-specific risk factors, primarily related to the immune-mediated pathogenesis of the disease, make SLE itself a clear, independent risk factor for CVD [10].

Despite recommendations to screen patients with SLE for CVD risk factors and to encourage healthy diets and exercise [11, 12], providers inconsistently screen [13]. Even when risk factors are identified, such as hypertension, diabetes, hyperlipidemia, and obesity, traditional interventions including behavioral modifications and medical management are insufficient to prevent CVD [13], which is unsurprising given the presence of non-classical, SLE-specific risk factors. Research and new therapies are needed to prevent premature atherosclerosis in pediatric patients with SLE.

Colchicine, known for its use in gout, Familial Mediterranean Fever (FMF), and other IL-1β mediated syndromes, has many anti-inflammatory and cardiovascular protective properties [14]. In particular, underscoring its utility in fever syndromes, colchicine has effects in inhibiting IL-1β activity via inhibition of the NALP3 inflammasome [14]. The Colchicine Cardiovascular Outcomes Trial (COLCOT) recently demonstrated that colchicine therapy provided significant benefit in prevention of secondary CVD for adults with previous MI [15], suggesting potential promise for CVD protection in other populations. Studies investigating colchicine’s potential role for cardiovascular protection are needed in children and adolescent patients with SLE. This article will review the current knowledge and background in therapy regarding cardiac atherosclerosis and coronary artery disease in childhood-onset and adult lupus, and will conclude by proposing investigation on the use of colchicine for prevention of CVD in young patients with SLE.

## Main text

### Role of inflammation in Atherosclerosis and CVD

All three components of the immune system – innate, cellular, and humoral immunity – are involved in the development of atherosclerosis. Differentiated macrophages upregulate receptors implicated in the innate immune response, including oxLDL and toll-like receptors [16]. Activation of these receptors results in the development of foam cells and the release of cytokines, vasoactive molecules, proteases, and oxidative species that contribute to plaque destabilization [17]. T-cells, in particular Th1 cells, also promote atherogenesis through their release of proinflammatory cytokines, including tumor necrosis factor (TNF), interferon-γ, platelet-derived growth factor, interleukin (IL)-1β, IL-2, IL-6, IL-8, and IL-12 [16, 18]. These cytokines and growth factors foster smooth muscle migration and proliferation, expanding the atherosclerotic plaque [19]. Autoantigens and autoantibodies, hallmarks of the humoral immune response, further contribute to atherosclerosis. Elevated levels of autoantibodies, including anti-oxLDL antibodies, anti-phospholipid antibodies, anti-cardiolipin antibodies, anti-β2GP1 antibodies, and anti-HSP antibodies, which contribute to endothelial damage and interrupt normal anti-atherosclerotic defenses, have been associated with atherosclerosis and thrombosis, and autoantigens within vessel walls and cholesterol molecules also play a role in inflammatory processes underlying atherogenesis [18].

Emerging evidence suggests that, of all these immunologic factors, IL-1β plays a primary role in the development of CVD [20–22]. Specifically, IL-1β promotes monocyte and leukocyte adhesion to vascular endothelium and development of atherothrombotic plaques [20–22]. In animal trials, blockade of IL-1β decreased atherosclerotic lesions and intimal thickening [23, 24]. Prior to the COLCOT trial, the Canakinumab Anti-inflammatory Thrombosis Outcome Study (CANTOS) took a definitive clinical step in highlighting the role of IL-1β by demonstrating that canakinumab therapy showed benefit in preventing secondary vascular events in adults after MI [25]. While complicated by a small but significant signal of fatal infection, conceptually this trial firmly established the role of IL-1β as a major driver of CVD [25].

### Increased risk of CVD in patients with SLE

Atherosclerosis is known to begin in adolescence even in the absence of SLE [26], yet patients with childhood-onset lupus experience accelerated atherosclerosis and early CVD [1, 2, 8]. Dyslipidemia is present in 63–85 % of children and adolescents with lupus even at initial diagnosis [27, 28], compared to approximately 20 % of the general pediatric population [29]. Despite premature atherosclerosis, clinical CVD manifested by MI and angina pectoris in pediatric patients with lupus is rare [4, 30, 31]. However, children with lupus do experience other acute cardiac diagnoses more commonly, with pericarditis being most common at about 10 %, followed closely by valvular insufficiency [32]. Myocarditis and endocarditis are less commonly reported in children [32]. It is possible that this early acute pericardial involvement and valvular insufficiency contribute to long-term CVD as the initial cardiac insults in some patients [33, 34].

As recognition of the high cardiovascular morbidity and mortality in young patients with SLE has grown, many studies have focused on the identification and understanding of factors associated with this increased risk. Analyses from the Atherosclerosis Prevention in Pediatric Lupus Erythematosus (APPLE) trial, during which patients underwent baseline measurements of carotid IMT (CIMT), demonstrate that increased CIMT is associated with both traditional and nontraditional CVD risk factors, including increased age, longer disease duration, minority status, higher body mass index, male sex, increased creatinine clearance, higher lipoprotein(a) level, proteinuria, azathioprine treatment, and prednisone dose [35].

### Iatrogenic risk factors

While sometimes overlooked, some of the medications used to reduce inflammation in SLE increase CVD risk by potentiating classical risk factors. Corticosteroids have many known adverse effects, including impaired glucose tolerance, hypertension, and obesity, which amplify these known traditional CVD risk factors. Azathioprine has also been associated with higher CIMT measurements in pediatric patients with SLE and increased rates of clinical CVD [35, 36], unlike the immunosuppressant agents mycophenolate mofetil and cyclophosphamide, which have not demonstrated significant impact on lipid profiles or CIMT [35, 37]. Conversely, hydroxychloroquine may be cardioprotective, by improving lipid profiles [38] and vessel elasticity [39]. This positive medication effect, however, is not enough to outweigh the many other factors that contribute to increased CVD risk in patients with lupus.

### Statins and the APPLE trial

Statins, which exert their effects through inhibition of 3-hydroxy-3-methyl-glutarylcoenzyme A (HMG-coA) reductase, the rate-limiting step in cholesterol synthesis, are first-line medications to treat hyperlipidemia and to prevent cardiovascular events in adults at risk of CVD. Statins also affect pathways related to inflammation and have many vasculoprotective effects [40], offering hope that they may prevent atherosclerosis in SLE, but studies in both adults and children with SLE have failed to show significant benefit [41, 42]. The Lupus Atherosclerosis Prevention Study (LAPS) of adult patients with SLE without clinical CVD found no difference in either the primary outcome (coronary artery calcium) or secondary outcomes (CIMT and carotid plaque) between the group treated with atorvastatin compared to placebo [41]. There was also no reduction in high sensitivity C-reactive protein (hsCRP) levels or any markers of endothelial activation, and patients who received atorvastatin experienced more liver toxicities than the control group.

Given that young patients have fewer comorbidities and traditional cardiovascular risk factors than adults and thus represent a unique population, the APPLE trial was designed to assess the use of statins in children and adolescents with SLE [42]. This multicenter, randomized, double-blind, placebo-controlled trial investigated the 3-year efficacy and safety of atorvastatin in preventing subclinical atherosclerosis progression in childhood-onset SLE. Although there was a general positive effect of treatment on slowing common CIMT in patients receiving atorvastatin, the results of this robust trial were not statistically significant, and thus routine use of statins in pediatric patients with lupus could not be supported. Unlike the LAPS in which atorvastatin was associated with liver toxicities, there was no concerning safety signal in children. Both the placebo and treatment groups experienced significant progression in CIMT over the 3-year period, and at a faster rate than that expected in the general population, supporting the understanding that subclinical atherosclerosis in patients with SLE begins in childhood. This highlights the critical need for routine screening and management of CVD risk factors in pediatric rheumatology practice.

A secondary analysis of the APPLE study was conducted to evaluate whether certain subgroups, categorized by duration of disease, pubertal status, and variables linked to cardiovascular risk and CIMT, would benefit from statin therapy [43]. In this analysis, post-pubertal status and higher hsCRP levels were associated with lower CIMT progression in patients treated with atorvastatin, and this effect was greatest in the combined post-pubertal and high hsCRP group. Although secondary analyses must be interpreted with caution, these findings suggest that a more personalized approach should be considered for CVD risk reduction in SLE, as certain high-risk cohorts may benefit from more aggressive treatment strategies, including statin therapy and potentially other cardiovascular-protective interventions.

### Colchicine: mechanism, role in CVD, and potential use in SLE

Colchicine, an alkaloid initially extracted from the *autumn crocus* plant, is an anti-inflammatory medication used for over 2000 years to treat gout [44]. While its pharmacologic mechanisms are not completely understood, colchicine’s primary mechanism is via its binding to tubulins, forming a complex that interferes with polymerization of microtubules, a key component of the cytoskeleton [14, 45]. Colchicine’s impact on microtubules results in multiple downstream effects on mitotic activity, malignancy, and inflammation. Its anti-inflammatory actions include interference with neutrophil chemotaxis, adhesion, and mobilization through microtubule polymerization [46, 47], and suppression of superoxide production from neutrophils [48]. Importantly, colchicine also inhibits NALP3 (also known as cryopyrin) inflammasomes, thereby inhibiting caspase-1 activation and subsequently preventing nuclear factor κB activation and the production of active IL-1β and IL-18 [49, 50]. IL-1β is involved in monocyte and leukocyte adhesion to vascular endothelial cells, growth of vascular smooth muscle cells, and coagulation induction, and it stimulates the downstream IL-6 receptor signaling pathway, which promotes expression of fibrinogen and plasminogen activator factor, two important thrombotic mediators [51–53]. Both IL-1β and IL-18 (a member of the IL-1 family) are key proinflammatory cytokines and predictive of future adverse cardiovascular events, offering potential for colchicine as a cardioprotective therapeutic intervention [51].

Colchicine also has anti-fibrotic and cardiovascular benefits. It has shown potential in preventing and even reversing both amyloidosis and endothelial dysfunction in patients with FMF [54], and it reduces intimal hyperplasia by down-regulating leukocyte vascular endothelial growth factor expression after angioplasty in dogs [55]. In rat pulmonary arterial hypertension models, it reduced smooth muscle cell proliferation, increased cell apoptosis, and reduced expression of TNF-α and NF-κB both alone and when combined with a vasodilator [56], and it demonstrated synergy with atorvastatin in improving endothelial function and reducing inflammation in rats with hyperlipidemia [57].

Colchicine is currently used to treat various inflammatory conditions, including gout, FMF, Behcet’s disease, and recurrent pericarditis. Pericarditis associated with lupus has traditionally been treated with corticosteroids, although colchicine demonstrated effectiveness and was well tolerated in a series of 10 cases of lupus with pericarditis [58]. Colchicine has also exhibited long-term safety in studies of children with FMF with minimal adverse effects. A minority of patients experience transient transaminitis and transient, dose-dependent diarrhea, which resolves with split doses and/or dose reduction [59, 60]. Multiple long-term studies have supported colchicine’s tolerability in children of all ages with FMF; in two studies, one with 153 patients and another with 350 patients, colchicine was not discontinued in any of the patients over a period of 4 years and 6–13 years, respectively [59, 61]. Because colchicine is metabolized in the liver via a cytochrome P450 3A4-dependent pathway [62], caution should be taken when co-administering colchicine with other medications, notably cytochrome P450 3A4 inhibitors [60, 62].

Given its anti-inflammatory properties and tolerable side effect profile, colchicine has recently been studied in the context of cardiovascular protection. In the Low-Dose Colchicine (LoDoCo) trial, colchicine reduced cardiovascular events in patients with stable coronary disease, although this initial trial was small and unblinded [63]. Building on the LoDoCo trial, the large, randomized, double-blind, placebo-controlled COLCOT trial was conducted to evaluate the effects of colchicine on cardiovascular events as well as its long-term safety profile in patients who had recently experienced a MI [15]. Among these patients, those who received colchicine at a dose of 0.5 mg daily had a significantly lower risk of ischemic cardiovascular events (predominantly strokes and urgent hospitalizations for angina leading to coronary revascularization) compared to the placebo group. The most common side effects experienced by both groups were gastrointestinal, although there was no statistical difference in any gastrointestinal-related adverse event, including diarrhea. Pneumonia, however, did show statistical significance as a serious adverse event, although this event was uncommon overall, seen in 0.9 % of patients in the colchicine group and 0.4 % of those in the placebo group.

### Potential approach for future study

The APPLE trial laid invaluable groundwork for understanding how to approach future study of CVD in pediatric patients with SLE. Lessons from this trial suggest that robust methods of detecting early and subclinical vascular inflammation are needed to ensure a reliable and definable end point for a clinical study. A recent study in healthy adult patients suggests that CVD progresses rapidly in the fifth decade of life [64]. This study somewhat alters our paradigm of CVD as a slowly progressive, lifelong process, but it also may imply that factors which arise in healthy individuals to promote CVD during this fifth decade are accelerated by SLE, and that it is possible to detect these factors by imaging and molecular methods. One potential approach for a future study is to define these factors with more precision through collaboration with the wider scientific community, and then utilize them as markers for accelerated CVD in our patient population. At the same time, preliminary, well-powered studies of the effect of colchicine in childhood-onset lupus, using a broad array of currently known markers of subclinical CVD, are also needed and will add to our understanding. Following these studies, a broader trial of colchicine in pediatric patients with SLE for the prevention of CVD, with identifiable and measurable endpoints that clearly allow us to measure benefit, should be a real possibility. Since morbidity and mortality from accelerated atherosclerosis is uncommonly seen in the pediatric population with SLE, these studies in pediatric patients should extend into adulthood.

Study design in the form of a multi-center, randomized, double-blind, placebo-controlled clinical trial would most robustly capture potential benefits. Drawing from lessons of previous trials, selecting post-pubertal patients and including young adults may allow investigators to capture patients most at risk for CVD progression and thus most likely to benefit from an intervention. These participants, followed over both the medium- and long-term, would be assessed with carotid IMT, investigational serum markers of endothelial dysfunction such as specific circulating cytokines [65] including IL-18 and IL-6, or endothelial function mediators such as endothelial nitric oxide synthase [66]. Longer-term follow up could measure coronary artery calcium scores and the emergence of major adverse cardiac events in study groups.

## Conclusions

Given colchicine’s clear anti-inflammatory benefits and effectiveness in secondary prevention of CVD in adults, this drug offers hope for prevention of CVD in children and adolescents with SLE, a known inflammatory condition with long-term effects on the heart and vasculature. To our knowledge, no studies have been conducted investigating colchicine’s potential for cardiovascular protection in pediatric patients with SLE. The need for prevention of CVD in our patients is nothing less than paramount. As pediatric rheumatologists, it is our duty to allow our patients to enjoy full and complete lives despite their disease, both as children and beyond.

## Data Availability

not applicable.

## Abbreviations

SLE: Systemic lupus erythematous
CVD: Cardiovascular disease
MI: Myocardial infarction
FMF: Familial Mediterranean Fever
COLCOT: Colchicine Cardiovascular Outcomes Trial
TNF: Tumor necrosis factor
IL: Interleukin
CANTOS: Canakinumab Anti-inflammatory Thrombosis Outcome Study
APPLE: Atherosclerosis Prevention in Pediatric Lupus Erythematous
CIMT: Carotid IMT
LAPS: Lupus Atherosclerosis Prevention Study
hsCRP: High sensitivity C-reactive protein
LoDoCo: Low-Dose Colchicine
